# The Importance of Impact Loading and the Stretch Shortening Cycle for Spaceflight Countermeasures

**DOI:** 10.3389/fphys.2019.00311

**Published:** 2019-03-22

**Authors:** Markus Gruber, Andreas Kramer, Edwin Mulder, Jörn Rittweger

**Affiliations:** ^1^ Human Performance Research Centre, Universität Konstanz, Konstanz, Germany; ^2^ Institute of Aerospace Medicine, German Aerospace Center (DLR), Cologne, Germany

**Keywords:** plyometric exercise, whole-body vibration, muscle, bone, mechanoadaptation

## Abstract

Pronounced muscle and bone losses indicate that the musculoskeletal system suffers substantially from prolonged microgravity. A likely reason for these detrimental adaptations in the lower extremity is the lack of impact loading and the difficulty to apply large loading forces on the human body in microgravity. The human body is well adapted to ambulating in Earth’s gravitational field. A key principle herein is the periodic conversion of kinetic to elastic energy and *vice versa*. Predominantly tendons and to a lesser extent muscles, bones and other tissues contribute to this storage and release of energy, which is most efficient when organized in the stretch-shortening cycle (SSC). During SSC, muscles, especially those encompassing the ankle, knee, and hip joints, are activated in a specific manner, thereby enabling the production of high muscle forces and elastic energy storage. In consequence, the high forces acting throughout the body deform the viscoelastic biological structures sensed by mechanoreceptors and feedback in order to regulate the resilience of these structures and keep strains and strain rates in an uncritical range. Recent results from our lab indicate, notably, that SSC can engender a magnitude of tissue strains that cannot be achieved by other types of exercise. The present review provides an overview of the physiology and mechanics of the natural SSC as well as the possibility to mimic it by the application of whole-body vibration. We then report the evidence from bed rest studies on effectiveness and efficiency of plyometric and resistive vibration exercise as a countermeasure. Finally, implications and applications of both training modalities for human spaceflight operations and terrestrial spin-offs are discussed.

## Introduction

**Figure 1:**
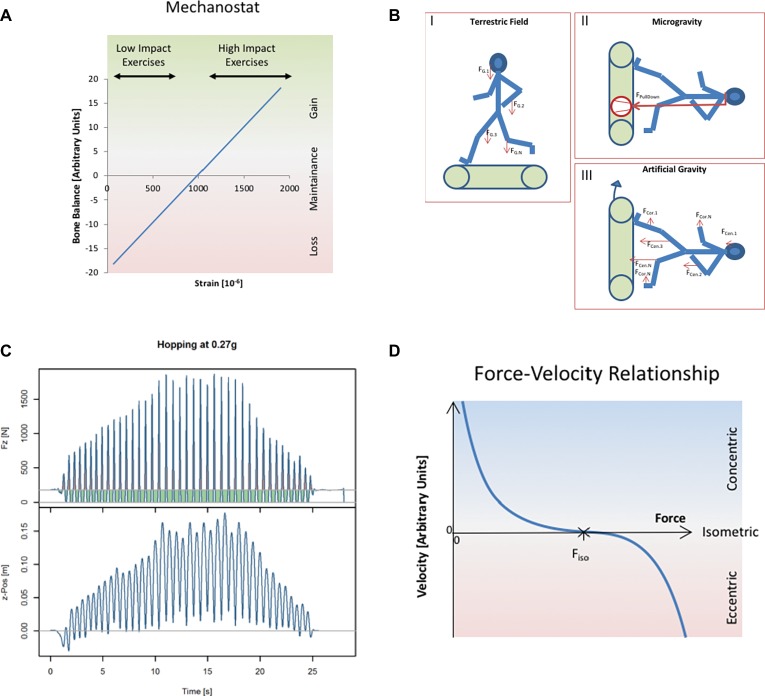
**(A)** Conceptualizationthe effect that tissue strains have on adaptive processes. This concept is well known in the bone field as “mechanostat” (Frost, 1987a), and it seems in principle to apply also to tendon, muscle, and likely other tissues. The mechanostat concept considers tissue’s mechanoadaption as a negative feed-back system, analogous to a thermostat. While thermostats enable constancy of temperature, the mechanostat keeps the tissue strains constant by adding or removing material in response to altering forces. Material evidence for the mechanostat concept had been provided by Rubin and Lanyon (1987), whose data are exemplified in the present diagram. Exercise-induced tibial compression strains are taken from Milgrom et al. (2000a,b) (low impact exercises, walking and bicycling; high impact exercises, running and drop jumps from different heights). **(B)** Different strategies to replace gravity by artificial pull-down forces. (I) Pull-down in the Earth’s gravitational field is a result of the body’s weight, which can be conceptualized as a homogeneous vector field within the entire body. (II) In microgravity, pull-down forces are generated by, e.g., pneumatic systems or bungees, and they are applied to the body *via* interfaces at the shoulders or hips. This “force concentration” is illustrated by size and thickness of the vector. (III) Artificial gravity replaces gravitational acceleration by centrifugal centrifugation. This avoids the problem of force application. However, it leads to in-homogeneity of the gravitational field (indicated here by different sizes of vectors) and generation of Coriolis forces, both of which become more pronounced with shorter radius of centrifugation. **(C)** Illustration of the time course of ground reaction forces during hopping, exemplified here for hopping during hypogravity simulation at *g* = 0.27 (first published in Weber et al., 2019). The vertical elevation of the body’s center of mass (z-position, lower panel) demonstrates that hops of increasing height are associated with increasing vertical ground reaction forces (Fz, upper panel). Green areas denote periods where Fz < weight (i.e., downward acceleration), and the red areas denote periods where Fz > weight (upward acceleration). Since no vertical net movement had occurred at the end of the hopping trial, the green and red areas are of equal size. Effectively, by fractioning the static load into “duty cycles,” one can produce ground contact forces that exceed the static loading force. The diagram therefore illustrates how jumping exercises can generate peak Fz that are substantially greater than static bodyweight. **(D)** The muscle’s working diagram, which also is known as “Hill’s” curve, is describing the force-velocity relationship of maximally activated muscle. In concentric mode, the muscle is shortening (*v* is positive) because the resistive force is smaller than the muscle’s contractile force. In isometric mode, resistive force and contractile force are equal, and *v = 0*. In eccentric mode, the resistive force exceeds the contractile force, and the muscle is lengthening (negative *v*). The force-velocity relationship is empirically described by a hyperbolic function (Hill, 1938).

Despite considerable amounts of crew time spent with countermeasure exercises on the International Space Station (ISS), astronauts still return to Earth with substantial musculoskeletal deficits (Rittweger et al., 2018), and these deficits are resilient to rehabilitation. One obvious explanation for the limited effectiveness of existing countermeasures is that subject loading forces on ISS are well below those on Earth. For example, foot and ground reaction forces are halved on the treadmill with vibration isolation and stabilization system (TVIS) that has been used on ISS since 2009 (Genc et al., 2010) and on the currently used T2 treadmill (De Witt and Ploutz-Snyder, 2014) when compared to running on Earth. This must lead to great reduction of musculoskeletal forces, and diminishment of tissue strains. However, sufficiently high strains and strain rates are a prerequisite for tissue maintenance in bone (Frost, 1987b) and tendon (Arampatzis et al., 2007). Likewise for muscle, costameric proteins are strain-sensitive, and their phosphorylation is impinging on anabolic and catabolic pathways (Fluck, 2006). Importantly, one has to understand that tissue adaptation is driven by the magnitude of tissue strains (see Figure 1A), and likely also strain rates (Mosley and Lanyon, 1998), and that, thereby, achieving loading forces of similar magnitude as on Earth, seem a prerequisite for full countermeasure effectiveness. One should consider also in this context that muscle contractions are crucially involved in the loading of the other skeletal structures (Schiessl et al., 1998; Rittweger, 2007; Yang et al., 2015).

The difficulty in achieving full loading forces in space is likely related to limitations in the external force that pulls the human body down toward the ground. While this pull-down force originates from gravitational attraction of the human body’s mass on Earth, it has to be generated artificially by, e.g., bungees or pneumatic cylinders. Very importantly, the artificially generated pull-down force has to be applied through interfaces such as shoulder pads, belts, etc. in microgravity (see Figure 1B). These interfaces can inflict pain and apprehension at higher loading forces, and it is therefore very difficult to train with a constant pull-down force in space that is equivalent to the body’s full weight.

With the pull-down force, the body can generate movements that involve specific time courses of loading, e.g., during jumping. Thus, the human body can generate ground reaction forces in excess of the magnitude of the primarily constant pull force. An example is illustrated in Figure 1C: despite a pull-down force of less than 250 N, peak ground reaction forces of more than 1,500 N are generated during hopping. Body movement is caused by muscle contractions within the human body, and modulation of ground reaction forces therefore is associated with modulation of muscle force and muscle length. Very importantly, muscles can generate greater forces when lengthening than when shortening (see Figure 1D). Optimal countermeasures for bone, tendon, and muscle therefore should involve active muscle stretching, which goes under the term “eccentric” contraction, simply because greater muscular forces can be generated than during isometric or concentric contraction. Since most of the human habitual activities, such as walking, running and jumping involve, alternating stretching and shortening of the muscle-tendon units, to the so-called “stretch-shortening cycles” (SSC), which enable storage and release of elastic energy, the anatomical and physiological features of the human organism are geared to optimize this important musculoskeletal function. In line with this understanding, recent data demonstrate that bone tissue strains are largest during hopping, followed by running and walking, and actually quite low during static exercises (Milgrom et al., 2000b). Hence, so-called plyometric training, which emphasizes stretch-shortening stimuli, constitutes an elegant strategy to enhance tissue strains when pull-down forces are limited. The current publication therefore ponders the countermeasure potential of reactive jumps as a physiological and of resistive vibration exercise as a technology-assisted way to elicit SSC.

## Physiology and Application of Reactive Jump Training

Already Cavagna et al. (1964) showed that while running, a substantial amount of energy is delivered at low cost. The mechanism underlying such a high movement efficiency has been described first by Goslow et al. (1973) in the cat hindlimb, and later on, Grillner (1975) proposed the SSC as a fundamental mechanism to increase power during natural movement tasks without extra energy expenditure. The SSC is not restricted to locomotion but constitutes a basic motor control scheme that is underlying many human movements, like throwing, kicking, and hitting. The fundamental principle is to store elastic energy in tendons, muscles, and other tissues. Running and hopping are ideal examples to illustrate how energy transformation helps to save metabolic energy and increase the economy of a movement. Thus, from an anatomical point of view, the Gastrocnemius-Achilles tendon complex, with its short and strong muscle and its long and compliant tendon seems an optimized structure for SSC function (e.g., exemplified in the anatomy and locomotion of the kangaroo).

To ensure proper elastic energy storage, the respective muscle has to be considerably stiffer than its tendon so that pronounced lengthening of the tendon can occur with only minimal muscle lengthening (Lichtwark et al., 2007). This is beneficial for the storage of elastic energy as the tendon undergoes considerable stretching without energy losses, whereas muscle lengthening would imply futile cross bride cycles within the contractile elements (Flitney and Hirst, 1978; Lichtwark and Wilson, 2005). In contrast to the stiffness of the tendon, the stiffness of the muscle can be modulated within a fraction of seconds over a wide range by increasing or decreasing the number of cross-bridges. Whereas size and length represent anatomical features for optimized tendon and muscle function, this modulation of muscle stiffness mainly depends on muscle activation, underpinning the crucial role of neural control during SSC tasks (Taube et al., 2012).

The motor command that activates the muscle in a specific manner determines whether it dissipates energy, e.g., during landing, or stores energy, e.g., during hopping. In this respect, tendon and muscle can be seen as a cooperative couple able to attenuate or amplify power during specific motor tasks (Roberts, 2016). Note that during SSC, the overall performance is determined by the work done during the phase while the muscle-tendon units shorten. Without the tendon, the force-velocity properties of the muscle would limit force and consequently work with an increase in shortening speed during fast movements like jumps. With a tendon that stretches during the eccentric phase and recoils at high speed during this phase, the muscle itself can shorten more slowly, enabling a higher force production, and when we assume a similar strain, an increased muscle work.

Hopping is an ideal example to illustrate the fundamental neuromechanics of the SSC. The muscle activity pattern that constitutes the SSC can be divided into four distinctive parts: the preactivity (PRE), the short latency response (SLR), the medium latency response (MLR), and the long latency responses (LLR1 and LLR2) (Taube et al., 2008). The leg extensor muscles (triceps surae and quadriceps femoris) must be activated before touchdown when impact loading starts (PRE). The timing and activation level during PRE are crucial to adjust the stiffness of the muscle and control the impact loading during the ground contact. After ground contact during the breaking phase, the muscle-tendon unit lengthens, mainly because the tendon lengthens and stores elastic energy. It has been shown that stretch reflexes act in concert with descending motor commands to ensure a high activation of the extensor muscles throughout ground contact (Taube et al., 2008; Zuur et al., 2010). When increasing jump height, besides stiffness, force control of the muscle becomes more and more important. For maximal jumps, this often results in maximal forces in the leg extensor muscles with the unique feature that maximal muscle force can be reached in a much shorter time period during the SSC when compared to a maximal isometric contraction, resulting in a much higher strain rate.

In conclusion, the prerequisite of an efficient SSC is a stiff muscle and a tendon that can lengthen (absorb energy) and shorten (recoil energy) during movement. In addition, it has been proposed that the transition time between muscle-tendon unit stretching and shortening need to be minimal to avoid dissipation of stored elastic energy as well as a decrease in muscle force prior to the concentric phase (Komi and Gollhofer, 1997). As a consequence, the SSC is most economical with short SSC durations of often less than 200 ms. In this regard, the mechanical and neural properties of the muscle-tendon unit and the sensorimotor control loops seems to be perfectly tuned for an efficient SSC.

An exercise mode that is particularly suitable to train the SSC is plyometric training. Plyometrics are usually employed to increase jump height and leg power in athletes. As jumps are the exercise mode with the highest power output (Davies, 1971), it is not surprising that plyometrics consistently improve maximal jump height and peak power, with medium to large effect sizes, depending on the type of jumping exercise and training duration (Markovic, 2007; Moran et al., 2018). Using more than 50 jumps per session as well as a combination of different types of jumps seems to be beneficial (de Villarreal et al., 2009). In addition to jumps, other types of fast movements such as short sprints and change-of-direction maneuvers seem to benefit from plyometrics (Slimani et al., 2016). Fewer studies assessed the effect of jump training on maximal leg strength, but a recent meta-analysis reported high effect sizes for maximal leg strength after plyometrics (0.97), although they were lower than when plyometrics were combined with strength training (de Villarreal et al., 2009).

Plyometrics also lead to extremely high bone strains, especially in the tibia (Milgrom et al., 2000a,b; Ebben et al., 2010), and thus constitute a strong stimulus for bone. Indeed, habitual athletes in high-impact sports such as triple jumping (Niinimäki et al., 2017), volleyball (Rantalainen et al., 2010), sprint running (Wilks et al., 2009), or fencing (Chang et al., 2009) tend to exhibit higher bone mass and strength than matched controls.

In participants subjected to 2 months of bed rest, plyometrics have been successfully used as an integrated countermeasure (Kramer et al., 2017b), preserving muscle mass of leg extensors, maximal leg strength, and peak power as well as tibial mineral density and content, whereas the inactive control group showed profound losses in these parameters (Kramer et al., 2017a, 2018). Moreover, the jump training was able to prevent most of the deteriorations in functional parameters such as balance control, gait and mobility (Ritzmann et al., 2018). Excluding breaks, the training group exercised for about 3 min per day on 6 days per week. On average, each session consisted of 4 × 12 countermovement jumps and 2 × 15 repetitive hops, with each jump performed with maximal effort, and with 30–60 s of rest in between sets. For a detailed description of the training program, see (Kramer et al., 2017b). The high-intensity nature of the training may have been responsible for the cardiovascular adaptations (Maggioni et al., 2018). Indeed, other studies using jumps and other bodyweight exercises as a low-volume, high-intensity type of training demonstrated the effectiveness of this exercise mode for improving maximal oxygen uptake, which is considered to be the net criterion for cardiovascular function (McRae et al., 2012). A recent study examined the effect of different rest intervals on the acute physiological responses to jump exercise (Kramer et al., 2019). The authors report that jumping can elicit up to 98% of maximal oxygen uptake capacity (V′O_2max_) and maximal heart rate, with up to 40% of the exercise session spent above 90% of V′O_2max_ and a lactate accumulation of up to 9 mmol/L, provided the rest intervals are less than a second in between jumps and less than 30 s in between sets. Thus, with adequate work interval durations and work to rest ratios, jumps seem to be suitable as a form of high-intensity interval training (HIIT). As it is the case with other types of HIIT, there are several ways to effectively program a jump HIIT session, but one of the most important parameter seems to be the total time spent above 90% of V′O_2max_. For a review, see Buchheit and Laursen (2013).

This strongly suggests potential for plyometrics as a countermeasure against inactivity-induced neuromuscular and musculoskeletal deteriorations. In addition, with sufficiently short rest intervals, jumps can be used as a form of high-intensity interval training, thus not only putting high demands on the musculoskeletal but also the cardiovascular system.

## Physiology and Application of Resistive Vibration Exercise

In contrast to plyometrics where a force accelerates the human body against the ground, the principle idea behind vibration exercise is the utilization of a machine-generated force at the feet to elicit the training stimulus. By using sinusoidal oscillations, the external force becomes predictable to the user, and the harmonic waveform avoids higher frequency components that would be destructive to material of human and machine interfaces.

Vibration exercise, for the purpose of strength and neuromuscular training, is typically done with vibration frequencies between 20 and 40 Hz, and with peak-to-peak amplitudes (*A*_p2p_) up to 12 mm. Mathematically, the peak acceleration (*a*_pik_) in the sinusoidal cycle is given by the following:

$$a_{\mathrm{pik}}=2\;.\;A_{p2p}\;.\;\;{\left(\pi \;.\;f\right)}^{2}$$

where *f* is the vibration frequency. Thus, for a typical constellation with vibration with frequency *f* = 25 Hz and amplitude *A*_p2p_ = 10 mm, *a*_pik_ amounts to 123.4 m/s^2^, or 12.6 g. It is important to note that such large accelerations can usually not be achieved with other types of exercise. Equally important, periods of great loading alternate with periods of low loading magnitude. Thereby, the rate at which phases of high and low ground reaction forces alternate is greater than during walking or hopping (see Figure 1C). Thus, from a pure perspective of force magnitude, SSC must be expected to occur also during vibration exercise. The question still arises whether SSCs are also possible with period cycles of milliseconds only. That question had been addressed by Cochrane et al. (2009) who measured muscle contractile tissue displacement by B-mode ultrasound along with modulation of EMG activity during acute whole-body vibration. The authors report modulation of tendon length and muscle contractile length, both of them phase locked to the movement of the vibration platform. That study therefore clearly demonstrates the presence of SSC at 6 Hz vibration frequency, and a current study in our lab ought to establish whether even higher vibration frequencies allow SSC in the exercising muscle.

Solid evidence is also available to demonstrate that vibration-induced SSC is associated with phase-locked modulation of electromyographic activity in the stretched muscles (Cochrane et al., 2009; Ritzmann et al., 2010), thus strongly suggesting the occurrence of stretch reflexes (Burke et al., 1976; Rittweger, 2010; Ritzmann et al., 2010), although this notion has been questioned (Cakar et al., 2015) and an alternative reflex pathway has been proposed (Karacan et al., 2017). Thus, future research should establish the exact nature of the reflex responses to vibration, and also the magnitude of the musculoskeletal force modulation during vibration-induced SSC. Most probably, these forces are much lower than the ones elicited by plyometrics, but what vibration lacks in force magnitude might be compensated by the very high number of repetitions. Keep in mind that even only 1 min of vibration training at 30 Hz translates to 1,800 cycles. When used in a bed rest study, whole-body vibration, applied twice daily at 20 Hz, with *A*_p2p_ ≤ 4 mm, with additional loads of 15% of body weight and no additional exercises was found ineffective to maintain both the musculature (Zange et al., 2008) and bone (Baecker et al., 2012). By contrast, in the 56-day Berlin Bed Rest (BBR) study, vibration was combined with resistive exercise (Rittweger et al., 2006). With a progressive overload protocol, subjects performed squatting exercise, heel raises, toe raises, and kicking exercise with a loading equivalent to twice the body weight in bouts of 60–100 s. This was performed 11 times per week, with inter-subject’ competition days on Wednesdays and resting days on Sundays. This training regimen could preserve muscle strength and power (Mulder et al., 2006; Buehring et al., 2011), prevent bone loss (Armbrecht et al., 2010; Rittweger et al., 2010), and mitigate spinal deconditioning (Belavy et al., 2008) and shrinkage of the femoral artery (Bleeker et al., 2005). In a follow-up study (Belavy et al., 2010), it was demonstrated that vibration *per se* can contribute to bone maintenance (Belavy et al., 2011), but no such effect could be demonstrated for muscle (Mulder et al., 2009). In conclusion, in the bed rest setting, resistive vibration exercise has demonstrated excellent effectiveness against musculoskeletal de-conditioning, and also some cardiovascular effectiveness. It is also noteworthy in this context that vibration exercise is increasingly used not only in rehabilitation medicine, foremost in the field of pediatrics, but also in geriatrics (Semler et al., 2008; Stark et al., 2010).

## Defining an Optimized Prescription Impact Loading for Space

**Figure 2:**
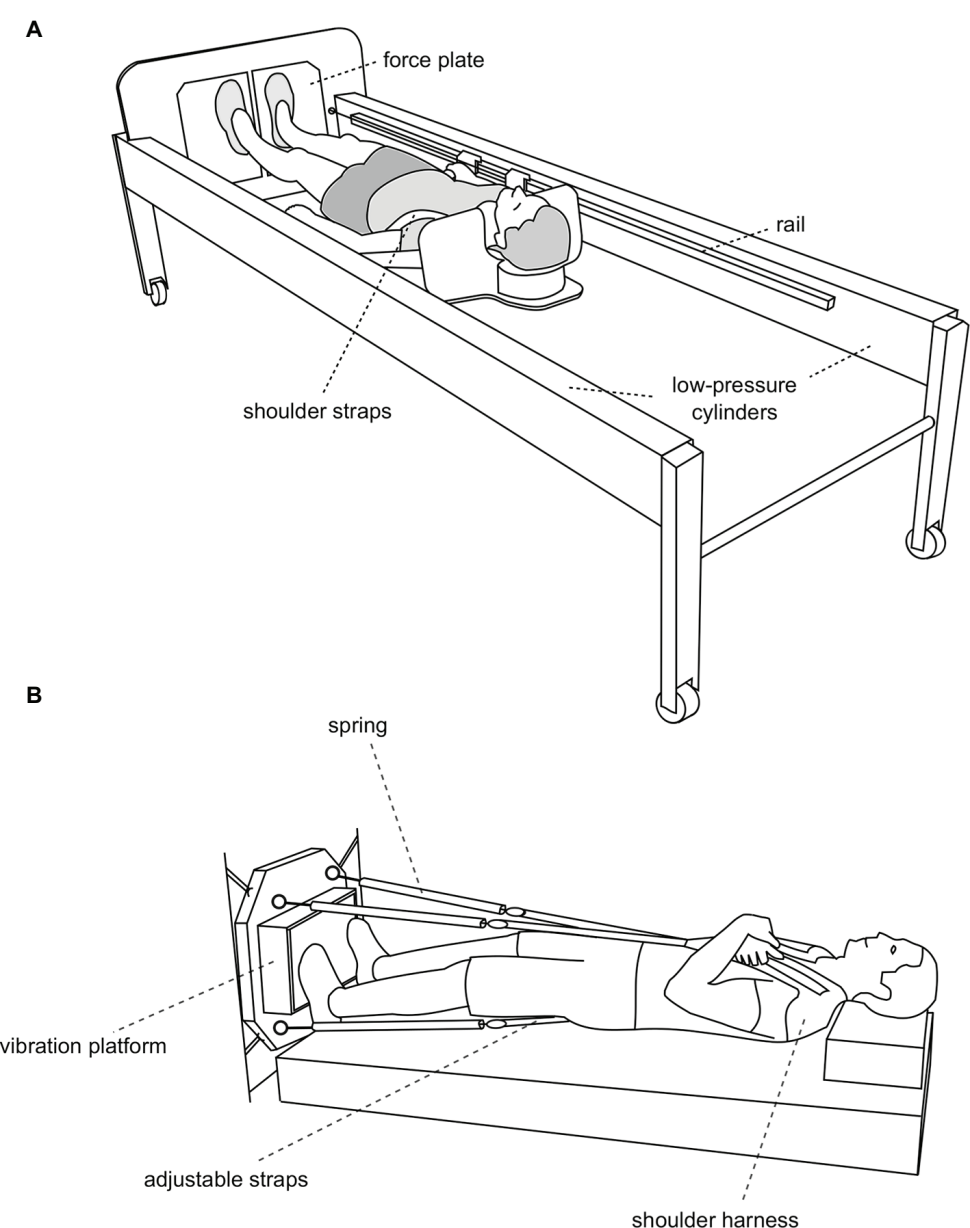
**(A)** The jump training device (sledge jump system, SJS, adapted from Kramer et al., 2017b). The participant is fixed to the sledge with shoulder straps, and his thighs rest on additional straps. The straps are attached to the rails and can slide alongside the rails with minimal friction. The forces generated by the two low-pressure cylinders substitute the gravitational force. Any force between 0 and 1,800 N can be set by altering the pressure of the cylinders. Details about the system can be found elsewhere (Kramer et al., 2010, 2012a,b). **(B)** Whole-body vibration (WBV) in microgravity (adapted from Kramer et al., 2013). The subject is pulled toward the vibration platform by four springs *via* four adjustable straps. The straps are adjusted in a way that the springs exert a force that matches the subject’s body weight.

In the aforementioned RSL bed rest study, the participants used a sledge jump system (SJS) to perform the jump training in a horizontal position. This system was designed for the use in microgravity, as it does not require gravitational forces (Figure 2A). Instead, the counterforce for the jumps is generated by low-pressure cylinders, allowing for forces above and below bodyweight to be used to adjust the training load (Kramer et al., 2012b). Before transferring this terrestrial concept to microgravity, the subject fixation in the SJS would have to be slightly altered, as the gravitational pull that stabilizes the subject during the jumps is lacking in microgravity. In addition, the feasibility of attaching the SJS to the spacecraft’s structure without transferring excessive forces has to be verified. Both of these modifications could be tested during a parabolic flight campaign.

The advantage of plyometric training is that it would be highly compliant with current ISS operations, as the training time would be much shorter than the currently scheduled two to two and a half hours per day. This would also be an advantage in future human exploration missions with restrictions on exercise time and minimal ground communication. However, the challenge would be to fit the SJS into a smaller spacecraft, as the minimum length even of a modified system would be the height of the astronaut plus his or her maximal jump height, i.e., for taller astronauts about 2.5 m.

If plyometrics were successfully used by astronauts as a short exercise countermeasure for the deconditioning of muscle, bone and also the cardiovascular system, this could have a major impact on the use of such a training program in sedentary populations, due to the astronauts’ function as role models. If a major part of the sedentary population would adopt such a short plyometric training, this might result in a pronounced decrease in sarcopenia, dynapenia, osteoporosis, and cardiovascular diseases.

Plyometric training could be complemented by resistive vibration exercise. This is particularly true where plyometric training is not possible due to operational constraints (e.g., mass upload, available room) or limited exercise capacity or willingness on the side of exercisers. In the first Berlin bed rest study, a platform with two excenter rotations in anti-phase was used that was specifically designed for that study (Rittweger et al., 2006). In the second Berlin bed rest study, a commercially available platform with directly driven oscillation was used (Belavy et al., 2010). In addition, whole-body vibration was already successfully tested during several parabolic flight campaigns (Kramer et al., 2013), see Figure 2B. It would be straightforward to implement flight models built on one of these already tested vibration systems.

With regard to exercise prescription, it must be expected that astronauts have to exercise at least once a day. This is based on the existing evidence that a total of 11 exercise sessions on 6 days of the week, as prescribed in the first Berlin bed rest study fully prevented bone loss and muscle weakening (Rittweger et al., 2010), but the performance of virtually the same training on 3 days only of the week was not enough to fully prevent bone loss (Belavy et al., 2011) and muscle weakening (Mulder et al., 2009). Ideally, exercises would be performed in bouts of 30–60 s, which leads to provenly high serum lactate levels, mimicking some of the physiological demands of many of the well-established “high intensity interval training” prescriptions.

## Author Contributions

MG and JR contributed structure of the work. MG, AK, and JR drafted the manuscript. All authors contributed to manuscript revision, read, and approved the submitted version.

### Conflict of Interest Statement

The authors declare that the research was conducted in the absence of any commercial or financial relationships that could be construed as a potential conflict of interest.
